# Supplementary material for: Bacteriophage-Resistant Salmonella rissen: An In Vitro Mitigated Inflammatory Response
Source: Viruses. 2021 Dec 9;13(12):2468. doi: 10.3390/v13122468 (PMC8703591; doi:10.3390/v13122468)
Supplement: Supplementary file 1 [file viruses-13-02468-s001.zip › viruses-1477427-supplementary.pdf]

Table S1. Primers used for RT-qPCR.

GENE	PRIMER
GAPDH (Glyceraldehyde-3-Phosphate Dehydrogenase)	FW: 5’-CCTCTGACTTCAACAGCGACAC-3’<br>RW: 5’-CACCACCCTGTTGCTGTAGCCA-3’
TLR4 (Tool like receptor 4)	FW: 5’-TGAAACCCAGAGCTTTCAGACTCC-3’<br>RW: 5’-GGAGGTTGTCGGGGATTTTG AG-3’
sspH1	FW: 5’-TGCAGAAAAAGGGGAATACG-3’<br>RW: 5’-GCAGCCTGAAGGTCTGAAAC-3’
sodC1	FW: 5’-TATTGTCGCTGGTAGCTG-3’<br>RW: 5’-CAGGTTTATCCGAGTAA-3’
gtgE	FW: 5’-AGGAGGAGTGTAAAGGT -3’<br>RW: 5’-GTAGAACTGGTTTATGAC-3’
spvC	FW: 5’-*ACTCCTTGCACAACCAAATGCGGA*-3’<br>RW: 5’-TGTCTTCTGCATTTCGCCACCATCA-3’
gtgB	FW: 5’-TGCACGGGGAAAACTACTTC-3’<br>RW: 5’-TGATGGGCTGAAACATCAAA-3’
InvA	FW: 5’-GTGAAATTATCGCCACGTTCGGGCAA-3’<br>RW: 5’-TCATCGCACCGTCAAAGGAACC-3’
gogB	FW: 5’-TGCAATCTGCCTGCACATATAG-3’<br>RW: 5’-CCCAGACCGCATCTGTTAATG-3’
RecA	FW: 5’-CCATGGATGGCTATCGACGAAAAC-3’<br>RW: 5’-TTCGAATTAAAAATCTTCGTTGG-3’
LpxR	FW: 5’-CGTTTTCCCCTGTAACGTGT-3’<br>RW: 5’-CGGACTGGAGTGGAATTCAT-3’

Table S2. Antibody used for Western blot.

**Primary Antibodies**	**Company**
β-Actina	Santa Cruz Biotechnology; mouse monoclonal antibody
pNFKB	Cell Signaling; rabbit monoclonal antibody
NFKB	Cell Signaling; rabbit monoclonal antibody
IKBα	Cell Signaling; rabbit monoclonal antibody
pSTAT3	Cell Signaling; rabbit monoclonal antibody
STAT3	Cell Signaling; rabbit monoclonal antibody
pAKT	Cell Signaling; rabbit monoclonal antibody
AKT	Santa Cruz Biotechnology; mouse monoclonal antibody

**Secondary Antibodies**	**Company**
Goat Anti-Rabbit HRP	Bio Rad
Goat Anti-Mouse HRP	Bio Rad

Table S3. Antibiogram of RW and RR strains. S: Sensitive; NI: Not Identified; R: Resistant.

**Antibiotics**	**R^W**	**R^R**
Ampicillin	S	S
Azithromycin	NI	NI
Cefepime	NI	NI
Cefotaxime	S	S
Cefotaxime/clavulanic acid	NI	NI
Cefoxitin	R	R
Ceftazime	S	S
Ceftazime/clavulanic acid	NI	NI
Cloramphenicol	S	S
Ciprofloxacin	S	S
Colistin	S	S
Ertapenem	S	S
Gentamicin	S	S
Imipenem	S	S
Meropenem	S	S
Nalidixic Acid	S	S
Sulfamethoxazole	NI	NI
Temocillin	NI	NI
Tetracycline	S	S
Tigecycline	S	S
Trimethoprim	S	S
